# A Test of Positive Association for Detecting Heterogeneity in Capture for Capture–Recapture Data

**DOI:** 10.1007/s13253-017-0315-4

**Published:** 2017-12-11

**Authors:** Anita Jeyam, Rachel S. McCrea, Thomas Bregnballe, Morten Frederiksen, Roger Pradel

**Affiliations:** 1 National Centre for Statistical Ecology, School of Mathematics, Statistics and Actuarial Science, University of Kent, Canterbury, CT2 7NF UK; 2 Department of Bioscience, Aarhus University, Rønde, Denmark; 3 Department of Bioscience, Aarhus University, Roskilde, Denmark; 4 Centre d’Ecologie Fonctionnelle et Evolutive UMR 5175, CNRS - Université de Montpellier - Université Paul-Valéry, Montpellier - EPHE, Montpellier, France

**Keywords:** Cormack–Jolly–Seber model, Goodman–Kruskal’s gamma, Goodness-of-fit

## Abstract

The Cormack–Jolly–Seber (CJS) model assumes that all marked animals have equal recapture probabilities at each sampling occasion, but heterogeneity in capture often occurs and should be taken into account to avoid biases in parameter estimates. Although diagnostic tests are generally used to detect trap-dependence or transience and assess the overall fit of the model, heterogeneity in capture is not routinely tested for. In order to detect and identify this phenomenon in a CJS framework, we propose a test of positive association between previous and future encounters using Goodman–Kruskal’s gamma. This test is based solely on the raw capture histories and makes no assumption on model structure. The development of the test is motivated by a dataset of Sandwich terns (*Thalasseus sandvicensis*), and we use the test to formally show that they exhibit heterogeneity in capture. We use simulation to assess the performance of the test in the detection of heterogeneity in capture, compared to existing and corrected diagnostic goodness-of-fit tests, Leslie’s test of equal catchability and Carothers’ extension of the Leslie test. The test of positive association is easy to use and produces good results, demonstrating high power to detect heterogeneity in capture. We recommend using this new test prior to model fitting as the outcome will guide the model-building process and help draw more accurate biological conclusions. Supplementary materials accompanying this paper appear online.

**Electronic Supplementary Material:**

Supplementary materials for this article are available at 10.1007/s13253-017-0315-4.

## Introduction

Capture–recapture models are widely used in ecology to estimate abundance, survival and/or movement probabilities. Several models can be fitted to a dataset, and model selection is usually performed based on information criteria such as the AIC, which select the least worst model amongst the candidate fitted models. Hence, absolute goodness-of-fit (GOF) assessment is crucial to ensure that the set of candidate models includes at least one model that provides an adequate fit to the data (Pradel et al. 2003). Furthermore, diagnostic tools targeting specific phenomena provide guidance as to the possible reasons for lack-of-fit, thus pointing towards better fitting models. Focussing on the simplest form of capture–recapture model for open populations: the Cormack–Jolly–Seber (CJS) model, we propose a new test to detect heterogeneity in capture, using Goodman–Kruskal’s gamma (Siegel and Castellan 1988).

Capture–recapture is a technique used to obtain data from animal populations: animals are captured, individually marked, released, then resighted or recaptured at different sampling occasions. When the parameter of interest is solely the survival probability, the information recorded for each animal is 0 (not seen) or 1 (seen) at each occasion. The CJS model can be fitted to the collected dataset. This model assumes equal recapture probabilities for all marked animals present at each sampling occasion. This assumption is violated when animals have intrinsically different capture probabilities, in which case the dataset exhibits heterogeneity in capture. For example, this may occur when there is a social structure within the population, or if some animals are in locations which are easier to observe than others.

This phenomenon is not routinely tested for, unlike trap-dependence (capture at a given occasion affecting capture probability at the following occasion) or transience (animals just passing through the study site), which are the object of existing test components used to assess the GOF of the time-dependent CJS model (Pradel et al. 2005). The modified version of Leslie’s equal catchability test (Orians and Leslie 1958) and Carothers’ extension of the Leslie test (Carothers 1971), which both target heterogeneity in capture do not seem to be widely used, perhaps due, respectively, to the amount of data discarded and the theoretical complexity of the test. We propose an alternative method to detect heterogeneity in capture, using Goodman–Kruskal’s gamma (Siegel and Castellan 1988), which is relatively simple to construct and apply.

It is important to identify and account for heterogeneity in capture when it occurs as not accounting for it can lead to biases in estimates of demographic parameters such as survival or abundance. Although survival estimates have been shown to be fairly robust, even small biases can lead to flawed inference or have an impact on management strategies (Prévot-Julliard et al. 1998; Cubaynes et al. 2010; Fletcher et al. 2012; Abadi et al. 2013). For example, Fletcher et al. (2012) and Abadi et al. (2013) both observed negative biases in the survival estimates when fitting models that did not account for heterogeneity in capture. It is also known that ignoring heterogeneity in capture leads to underestimating abundance, whether the framework is closed or open populations (see for example Morgan and Ridout 2008; Cubaynes et al. 2010; Pledger et al. 2010).

Furthermore, the presence of heterogeneity in capture can reveal biological insights. Indeed, investigations as to its causes may be warranted, which in turn may lead to identifying individuals with different behavioural patterns such as breeders/non-breeders, bold/timid, dominant/subordinates, or animals with different feeding strategies (Corkrey et al. 2012). The sampling regime in combination with the occurrence of heterogeneity can give clues to which behavioural pattern could be involved.

Finally, heterogeneity in capture can also be a result of the study design (Oliver et al. 2011; Corkrey et al. 2012), and identifying it would give directions to possible adjustments. Indeed, heterogeneity in capture can be related to the sampling process, or stem from resighting errors, particularly for rings or neck-bands read at large distances, in which case additional data collection rules will be specified: for example, it is more or less standard practice to require at least two observations for neck-banded geese (Madsen et al. 2014).

The presence of heterogeneity in capture will be identified by a significant test result. Following this, models accounting for this feature should be fitted as part of the candidate model set. Some possible techniques to incorporate heterogeneity in capture are: using observed covariates for modelling the capture probability, using a latent structure: finite mixture models, (Pledger et al. 2003) or hierarchical classes of animals with proportional capture probabilities (Pradel et al. 2010; Oliver et al. 2011); Corkrey et al. (2012) provide a method to incorporate heterogeneity in capture in a Bayesian framework.

The tests derived in this paper are motivated by a dataset of Sandwich terns (*Thalasseus sandvicensis*), collected between 2003 and 2012 by the National Environmental Research Institute at Aarhus University together with the Copenhagen Bird Ringing Centre at the Danish Zoological Museum. The study took place on Hirsholm, a 15 ha inhabited island in northern Kattegat, Denmark (7 km NE of Frederikshavn; $57^\circ 29^\prime N-10^\circ 37^\prime$E). One of the goals of the study was to estimate survival in order to assess whether it increased after the introduction of a control programme of large gulls through culling in 2007. These gulls predated on breeding adult Sandwich terns as well as their eggs and chicks. Heterogeneity in capture was suspected in this population, and we applied our tests to formally identify it. We then fitted simple CJS models with and without heterogeneity in capture, to illustrate the impact on the survival estimates.

The paper is structured as follows. The new test of positive association and the other tests examined are described in Sect. 2. In Sect. 3, we assess the performance of the test of positive association relative to the alternative tests using simulation. The Sandwich tern study is then presented in Sect. 4. The paper concludes with a discussion and recommendations in Sect. 5.

## Tests for Detecting Heterogeneity in Capture

### A New Test of Positive Association

We propose a test of positive association using Goodman–Kruskal’s gamma as a way of detecting heterogeneity in capture. If some animals have a higher capture probability than others, they will be seen more often. In such a case, at a given capture occasion, animals with a high number of previous encounters will likely have a high number of future encounters. We use a toy example comprising three capture histories (see Table 1) and focus on occasion $i=5$ to illustrate the steps of the test.

We propose the following steps to construct the test statistic of interest to our objective. Firstly, the test should target heterogeneity in capture and therefore should not be contaminated by noise due to deaths or permanent emigration. Hence, the occasions after the last sighting, for which the presence of the animal is uncertain, are not included in the test construction. Likewise, since the CJS model conditions on first capture, the period prior to the first capture and the first capture occasion itself are not informative; thus these occasions are not incorporated. The last capture occasion itself does not provide any information to discriminate between the animals in terms of capture intensity and isn’t included in the test construction either. Thirdly, the occasions of first and last capture can differ amongst animals, leading to an artificial difference between them: the earlier (later) the animals are first (last) seen, the more possible encounters they have. Therefore, the information relative to the encounters is standardised by dividing the number of previous (future) encounters by the maximum number of possible previous (future) encounters. Fourthly, the raw proportions of previous and future encounters per animal at a given occasion are not of interest per se. Rather, we are interested in how animals fare relatively to one another: are animals that are seen more (less) often before *i* also seen more (less) often after *i*? Therefore, the ranks of these proportions constitute the final information retained from the data to test for heterogeneity in capture. Finally, since the range of ranks is limited and that we expect many ties, Goodman–Kruskal’s gamma is used to test for a positive association between the ranks of previous and future encounters (Siegel and Castellan 1988, p. 291). Since the test is based on previous and future encounters with respect to a given capture occasion *i*, it is reasonable to require a minimum of two informative occasions (i.e. excluding the first and last sightings) both before and after *i*. As a result, the test is restricted to animals known to be alive at least at $i+3$ and released before $i-1$; so it can only be computed for capture–recapture experiments with at least six capture occasions and performed from occasion 3 to $K-3$. Note that the capture history information at occasion *i* could be counted in either the future or previous encounters; since there is no strong argument in favour of either side we decided to count it in the previous encounters.

In our example, animal ID 98 is not used within the test: it is released before occasion 4, but never seen again so not known to be alive at occasion 8. The numbers of previous and future encounters (denoted *m*), as well as the proportions *pr*, are shown in Table 1 for our example animals ID 99 and ID 100.

**Table 1:** A toy example for extracting the information required for the test of positive association: for the test per occasion, at occasion $i=5$ and for the global test.

	Capture history										Previous encounters				Future encounters			
*Test of positive association per occasion*																		
Occasion *i*	1	2	3	4	**5**	6	7	8	9	10	*m*	*max*	***pr***	***rank***	*m*	*max*	***pr***	***rank***
ID 98	0	1	0	0	**0**	0	0	0	0	0	Not taken into account for test (not known to be alive at occasion 8)							
ID 99	1	0	0	1	**1**	1	1	1	0	0	2	4	**2/4**	**2**	2	2	**2/2**	**2**
ID 100	0	0	1	0	**0**	0	1	0	1	0	0	2	**0/2**	**1**	1	3	**1/3**	**1**
*Global test of positive association*																		
Occasion *i*	1	2	3	4	5	6	7	8	9	10	*m*	*max*	***pr***	***rank***	*m*	*max*	***pr***	***rank***
ID 98	0	1	0	0	0	0	0	0	0	0	global test of positive association not applicable							
ID 99	1	0	0	**1**	1	1	1	1	0	0	1	3	**1/3**	**2**	3	3	**3/3**	**2**
ID 100	0	0	1	0	0	**0**	1	0	1	0	0	3	**0/3**	**1**	1	2	**1/2**	**1**

For the test per occasion, the occasion of interest (here *i*=5) is denoted in bold. For the global test, the middle occasion is denoted in bold. *m* denotes the number of encounters, *max* the maximum possible number of encounters, and *pr* the proportion.

The gamma measure is estimated, based on the pairs of discordant *D* and concordant *C* observations: $\hat{\gamma }=\frac{C-D}{C+D}$. A pair of observations is concordant if the observation ranking higher (lower) for the previous encounters, also ranks higher (lower) for the future encounters; and discordant if the observation ranking higher (lower) for the previous encounters ranks lower (higher) for the future encounters. In our example from Table 1, animal ID 99 is ranked higher than animal ID 100 for both previous encounters and future encounters. Thus, animals ID 99 and ID 100 form a concordant pair. Animals who are ranked the same for either previous encounters or future encounters are not informative with regards to our objective. They form ties and are not taken into account by the gamma measure. In the case of heterogeneity in capture, we expect a high number of concordant pairs. Hence, we present the results of a one-sided test for $\gamma >0$.

The test statistic is constructed based on the asymptotic variance derived by Brown and Benedetti (1977), $\frac{\sum _{i}\sum _{j}v_{ij}(A_{ij}-D_{ij})^{2}-(4(C-D)^2/n)}{(C+D)^{2}}$, where $v_{ij}$ denotes the frequency cell from the contingency table *v* of rank of previous encounter proportions $\times$ rank of future encounter proportions, $A_{ij}=\sum _{k<i}\sum _{l<j}v_{kl}+\sum _{k>i}\sum _{l>j}v_{kl}$ and $D_{ij}=\sum _{k>i}\sum _{l<j}v_{kl}+\sum _{k<i}\sum _{l>j}v_{kl}$; *n* denotes the number of animals *n* used for the test. Note that since the test is performed at each occasion on a subset of animals, *n* is smaller than the original sample size.

When *n* is relatively large, under the null hypothesis of no association, the distribution of the test statistic $\frac{\hat{\gamma }}{\sqrt{\hat{{Var}(\gamma )}}}$ is approximately a standard normal (Siegel and Castellan 1988). In order to be conservative regarding this approximation, we propose to restrict *n* to at least 30. If $n<30$, we state that the test is Non Applicable, which we denote by NA.

The subsets of animals used for this test at different occasions *i* are not independent, which means the results from each occasion cannot be pooled. However, in a situation where not much temporal variation is expected for the capture probability, one may use a global version of the test instead. This global test is based for each animal, on the occasion allowing for the best balance between information brought by previous and future encounters, that is the middle occasion between first and last capture. The test procedure and restrictions are the same as the test for a given occasion *i*, only *i* will be replaced by the middle occasion and each animal used only once within the test. The global test of positive association is illustrated for our toy example in Table 1.

### Alternative Tests

#### Use of Existing Diagnostic GOF Tests

The existing diagnostic GOF tests currently implemented in U-CARE (Pradel et al. 2003; Choquet et al. 2009a) are based on Chi-square contingency table tests and are formed of four components. Tests 2.CT and 3.SR have directional components that are, respectively, used to detect short-term trap-happiness/shyness (i.e. directional trap-dependence) and transience (animals just passing through the sampling site and therefore likely to be caught only once). Components 3.Sm and 2.CL have a less straightforward interpretation, although Test 2.CL is thought to indicate a long-term trap-dependence effect. When the data are sparse for 3.Sm and/or 2.CL, the cells within the corresponding tables are pooled (this does not occur for components 2.CT and 3.SR, which are based on $2 \times 2$ tables). If the data are still sparse after pooling, Fisher’s exact test is used. The diagnostic goodness-of-fit tests are NA when the contingency tables have a row or column total of zero. The sum of the four GOF components forms the classic omnibus Chi-square test statistic (McCrea and Morgan 2014, Chapter 9). Tests 2.CT and 3.SR tend to generate significant results when there is heterogeneity in capture, but do not provide a diagnostic to differentiate a combination of trap-dependence and transience from heterogeneity in capture. Péron et al. (2010) suggest using corrected test statistics obtained by removing the squared directional components of Test 3.SR and 2.CT from the overall Chi-square statistic in order to assess the goodness-of-fit of a mixture model accounting for heterogeneity in capture.

#### Leslie-Carothers Test of Equal Catchability

The Leslie test of equal catchability tests whether the sampling of marked animals is non-random. It is based on the frequency of recaptures, within groups of animals with the same first release occasion and the same last capture occasion (it is used for capture histories with a minimum of 5 occasions). We use a modified version of Leslie’s test, corresponding to Cochran’s *Q*, presented in Carothers (1971), which has a proven asymptotic distribution, keeping the original sample size recommendation of at least 20 animals per group (Orians and Leslie 1958). The results are presented pooled by first release occasion (if there is at least a non-missing test result for one of those groups, otherwise the pooled test is NA).

Carothers has further extended Leslie’s approach by providing a more efficient test that uses more data than the Leslie test. The resulting test statistic follows a Chi-square distribution under the null hypothesis of equal recapture probability for all marked animals known to be alive and is not partitioned by first capture occasion, unlike the Leslie test (see Carothers (1971) for further details).

## Simulation Study

A subset of the different scenarios simulated to investigate the methods considered are shown in Table 2: $p_{1}$, $p_{2}$, $\phi _{1}$ and $\phi _{2}$, respectively, denote the capture and survival probabilities in groups 1 and 2; $\pi _{1}$ denotes the proportion of individuals in group 1. Our basic heterogeneity scenarios had two classes of animals with contrasting capture probabilities of 0.35 and 0.82 and proportion of 0.3 for one or the other class. These encounter probabilities are roughly based on some of the estimates obtained for highly and poorly detectable wolves in Cubaynes et al. (2010). In order to assess the test properties in good conditions, survival probability was set to 0.9. Based on the discrete heterogeneous capture scenarios, denoted by HC1 and HC2, we also simulated discrete heterogeneity with slight time variation, by adding a uniform term U[-0.20,0.17] to the original capture probabilities at each time-point; we denote these by HC1t and HC2t. We also considered different cases of continuous heterogeneity in capture, with the capture probability, *p*, following a beta distribution:

- HCc1: symmetric around the mean, generated by a $\beta (5,5)$: mean 0.5 and standard deviation (sd) 0.15.
- HCc2: positive skew (most animals with low capture probabilities), generated by a $\beta (4,12)$: $\mathrm{mean}\,\mathrm{(sd)}=0.25\,(0.11)$
- HCc3: negative skew (most animals with high capture probability), generated by a $\beta (12,4)$: $\mathrm{mean}\,\mathrm{(sd)}=0.75\,(0.11)$.
- HCc1F: symmetric around the mean, generated by a $\beta (2,2)$: $\mathrm{mean}\,\mathrm{(sd)}=0.50\,(0.22)$.
- HCc2F: positive skew (most animals with low capture probabilities), generated by a $\beta (2.4,4.3)$: $\mathrm{mean}\,\mathrm{(sd)}=0.36\,(0.17)$.
- HCc3F: negative skew (most animals with high capture probability), generated by a $\beta (4.3,2.4)$: $\mathrm{mean}\,\mathrm{(sd)}=0.64\,(0.17)$.

The types of heterogeneity scenarios considered for *p* are illustrated in Web Figure 1 and quantile tables given in Web Table 1. For all our scenarios of heterogeneity in capture, movement between groups was not allowed. These various parameter values were examined in order to assess the power of the test under different scenarios.

**Table 2:** Parameter values for simulation scenarios considered: $p_{1}$, $p_{2}$, $\phi _{1}$ and $\phi _{2}$, respectively, denote the capture and survival probabilities in groups 1 and 2, $\pi _{1}$ denotes the proportion of individuals in group 1. $\phi _{a1}$ denotes survival of newly marked animals, $\phi _{a2}$ the survival of previously marked animals. $p_\mathrm{TA}$ and $p_\mathrm{NTA}$ denote the probability of capture of a trap-aware and non-trap-aware animal (an animal is trap-aware at a given occasion *i* if it has been captured at $i-1$).

Scenario	$p_{1}$	$p_{2}$	$\phi _{1}$	$\phi _{2}$	$\pi _{1}$	$\phi _{a1}$	$\phi _{a2}$	$p_\mathrm{TA}$	$p_\mathrm{NTA}$
*Control for Type I error assessment*									
C1	0.35	0.35	0.9	0.9	–	–	–	–	–
C2	0.82	0.82	0.9	0.9	–	–	–	–	–
*Heterogeneous capture (2 groups) for power assessment*									
HC1	0.35	0.82	0.9	0.9	0.3	–	–	–	–
HC2	0.35	0.82	0.9	0.9	0.7	–	–	–	–
*Specificity assessment*									
Heterogeneous survival (2 groups) (HS)	0.9	0.9	0.45	0.9	0.3	–	–	–	–
Trap-shyness (TS)	–	–	0.9	0.9	–	–	–	0.62	0.82
Trap-happiness (TH)	–	–	0.9	0.9	–	–	–	0.55	0.35
Transience (TR)	0.82	0.82	–	–	–	0.4	0.9	–	–
Trap-shyness & transience (TSTR)	–	–	–	–	–	0.4	0.9	0.62	0.82
Trap-happiness & transience (THTR)	–	–	–	–	–	0.4	0.9	0.55	0.35

In order to assess the specificity of the test to heterogeneity in capture, trap-dependence, transience and heterogeneity in survival were also considered. The scenarios of short-term trap-dependence and transience are denoted by TH (trap-happiness) and TS (trap-shyness) and TR, respectively. We use the notation: $\phi _{a1}$ the survival of newly marked animals, $\phi _{a2}$ the survival of previously marked animals and lastly, $p_\mathrm{TA}$ and $p_\mathrm{NTA}$ denote the probability of capture of a trap-aware and non-trap-aware animal (an animal is trap-aware at a given occasion *i* if it has been captured at $i-1$). The heterogeneity in survival scenario is denoted by HS and consists of two groups of animals with equal capture probability, but one with low survival probability. The parameter values used are detailed in Table 2. Finally, we considered a combination of trap-dependence and transience scenario, denoted by THTR for trap-happiness and TSTR for trap-shyness.

We also considered control datasets (C1 and C2) with homogeneous and constant *p* and $\phi$ in order to check the Type I error rate obtained.

**Table 3:** Percentage of significant results, test of positive association per occasion using Brown and Benedetti’s asymptotic variance and 2 informative occasions, $N=2000$, high percentage of significant results in bold (> 50%).

Capture occasion	3	4	5	6	7
C1	4.40	3.60	6.80	6.40	4.80
C2	4.00	6.00	2.80	4.40	3.20
HC1	**98**.**00**	**100**.**00**	**100**.**00**	**100**.**00**	**100**.**00**
HC2	**100**.**00**	**100**.**00**	**100**.**00**	**100**.**00**	**100**.**00**
HC1t	**98**.**80**	**100**.**00**	**100**.**00**	**100**.**00**	**100**.**00**
HC2t	**100**.**00**	**100**.**00**	**100**.**00**	**100**.**00**	**100**.**00**
HCc1	**76**.**40**	**90**.**80**	**98**.**40**	**98**.**80**	**94**.**40**
HCc2	37.20	**56**.**00**	**59**.**60**	**58**.**80**	44.80
HCc3	**51**.**20**	**76**.**40**	**86**.**80**	**89**.**20**	**82**.**80**
HCc1F	**99**.**20**	**100**.**00**	**100**.**00**	**100**.**00**	**100**.**00**
HCc2F	**82**.**80**	**98**.**40**	**100**.**00**	**98**.**80**	**97**.**60**
HCc3F	**94**.**00**	**98**.**80**	**100**.**00**	**100**.**00**	**100**.**00**
HS	6.40	1.60	5.20	6.80	4.80
TS	1.20	0.40	0.00	0.00	0.00
TH	19.20	30.40	34.00	45.20	44.00
TR	7.60	5.60	2.40	5.20	4.80
TSTR	2.00	2.80	0.40	0.80	0.80
THTR	11.69 (248)	18.00	24.00	23.20	28.00

The number of applicable tests is 250 unless stated otherwise (indicated within brackets next to relevant number).

For each scenario, 250 datasets of 2000 and 500 animals, with 10 capture occasions, were simulated. Some representative results are presented for the simulations with 2000 animals, whilst additional results for the smaller sample size of 500 animals are provided in the supplementary Web material. For all the simulations, the results presented are the percentage of significant test results (out of the number of cases where the test was applicable), using a 5% level.

### Test of Positive Association

The results obtained from both the test of positive association per occasion and the global test are shown in Tables 3 and 4. Additional results are given in Web Tables 2 and 3 for a smaller sample size of 500.

The test per occasion shows very high power at all occasions for situations with discrete heterogeneity in capture and scenarios of continuous heterogeneity when the standard deviation is large (i.e. when heterogeneity is strong). It also has good power under these criteria for smaller sample size (see Web Table 2). As expected, it is less powerful for the scenarios of continuous heterogeneity with a smaller standard deviation. The test is not sensitive to trap-shyness, but it is sensitive to short-term trap-happiness and, hence, also to the scenario THTR which includes this phenomenon. Importantly, the test does not react to transience or heterogeneity in survival. The global positive association test using the middle occasion shows similar results: very high power for detecting heterogeneity in capture (including all scenarios of continuous heterogeneity), and also sensitivity to trap-happiness. It does not react to heterogeneity in survival, transience, or trap-shyness. The global test also retains good power for detecting heterogeneity in most of the scenarios, for a smaller sample size (see Web Table 3).

**Table 4:** Percentage of significant results, global test of positive association, using Brown and Benedetti’s asymptotic variance and 2 informative occasions, $N=2000$, high percentage of significant results in bold (> 50%).

Scenario	%
C1	4.80
C2	4.80
HC1	**100**.**00**
HC2	**100**.**00**
HC1t	**100**.**00**
HC2t	**100**.**00**
HCc1	**100**.**00**
HCc2	**75**.**20**
HCc3	**91**.**60**
HCc1F	**100**.**00**
HCc2F	**100**.**00**
HCc3F	**100**.**00**
HS	5.60
TS	0.00
TH	**65**.**20**
TR	3.60
TSTR	0.80
THTR	37.60

The number of applicable tests is 250.

### Diagnostic Goodness-of-Fit Components

The results obtained using the existing diagnostic GOF components (3.SR, 2.CT, 3.Sm, 2.CL) and total Chi-square (denoted Total), as well as the corrected tests (denoted 3.SRC, 2.CTC and TotalC), are presented in Table 5 and Web Table 4. Unlike the trap-dependence and transience phenomena, which have a clear-cut impact on Test 2.CT and 3.SR, respectively, heterogeneity in capture seems to affect all of the GOF components. For the scenarios considered, components 3.Sm and 2.CL seem to be impacted specifically in the case of heterogeneity in capture; however, they have only low power for datasets with 500 animals (see Web Table 4), and 2.CL even has low power for scenario HC2, with 2000 animals. Based on the simulated scenarios, the corrected approach suggested by Péron et al. (2010) is inconclusive. Because the tests did not have a particularly high power for detecting discrete heterogeneity in capture, scenarios of continuous heterogeneity were not considered here.

**Table 5:** Existing GOF components and corrected tests, $N=2000$ animals, percentage of significant results, high percentage of significant results in bold (> 50%).

Scenario	3.SR	2.CT	2.CL	3.Sm	Total	3.SRC	2.CTC	TotalC
C1	5.60	5.20	6.80	4.40	5.20	6.00	4.40	5.20
C2	6.80	4.40	2.00	4.40	4.00	6.00	4.00	3.20
HC1	**58**.**80**	**100**.**00**	**84**.**00**	**67**.**20**	**100**.**00**	10.40	21.20	**95**.**20**
HC2	**76**.**80**	**100**.**00**	18.80	**68**.**00**	**100**.**00**	15.60	22.00	**71**.**20**
HS	**100**.**00**	4.40	0.80	4.80	**100**.**00**	27.20	4.00	11.60
TS	6.00	**100**.**00**	5.20	4.40	**100**.**00**	6.00	26.80	12.00
TH	5.20	**100**.**00**	6.40	6.40	**100**.**00**	6.40	30.80	14.40
TR	**100**.**00**	2.40	0.00	4.00	**100**.**00**	**93**.**60**	3.20	**56**.**00**
TSTR	**100**.**00**	**100**.**00**	2.00	5.20	**100**.**00**	**96**.**00**	11.20	**62**.**00**
THTR	**100**.**00**	**100**.**00**	6.00	4.40	**100**.**00**	28.00	16.40	21.60

The number of applicable tests is 250.

### Leslie’s Test of Equal Catchability

The results obtained with the modified version of the Leslie test are shown in Table 6 and Web Table 5.

The results obtained show the test is very powerful for detecting heterogeneity in capture for $N=2000$, but it is also very sensitive to trap-happiness. Also, it is impractical to use for smaller datasets, since the number of applicable tests is most often null or low (see Web Table 5). This test is not sensitive to trap-shyness, heterogeneity in survival or transience.

**Table 6:** Modified Leslie’s test, $N=2000$ animals, percentage of significant results (number of applicable tests), high percentage of significant results in bold (> 50%).

1st release occasion	1	2	3	4	5	6
C1	3.21 (249)	2.40 (250)	4.00 (250)	2.40 (250)	2.00 (250)	1.60 (250)
C2	1.60 (250)	4.80 (250)	2.40 (250)	2.00 (250)	2.40 (250)	1.20 (250)
HC1	**99**.**20** (250)	**99**.**20** (250)	**99**.**60** (250)	**97**.**20** (250)	**90**.**80** (250)	**70**.**80** (250)
HC2	**100**.**00** (250)	**99**.**60** (250)	**100**.**00** (250)	**99**.**60** (250)	**99**.**60** (250)	**86**.**00** (250)
HC1t	**99**.**60** (250)	**100**.**00** (250)	**98**.**00** (250)	**97**.**60** (250)	**95**.**20** (250)	**74**.**40** (250)
HC2t	**100**.**00** (250)	**100**.**00** (250)	**100**.**00** (250)	**99**.**60** (250)	**96**.**80** (250)	**80**.**40** (250)
HCc1	**85**.**60** (250)	**82**.**80** (250)	**74**.**40** (250)	**60**.**80** (250)	42.40 (250)	22.80 (250)
HCc2	36.06 (208)	31.12 (241)	28.11 (249)	22.80 (250)	17.60 (250)	8.00 (250)
HCc3	**70**.**80** (250)	**63**.**60** (250)	**58**.**00** (250)	47.20 (250)	27.60 (250)	18.00 (250)
HCc1F	**98**.**80** (250)	**99**.**20** (250)	**99**.**20** (250)	**97**.**60** (250)	**91**.**20** (250)	**58**.**80** (250)
HCc2F	**87**.**45** (247)	**88**.**00** (250)	**82**.**00** (250)	**70**.**00** (250)	48.40 (250)	23.60 (250)
HCc3F	**96**.**80** (250)	**98**.**00** (250)	**96**.**80** (250)	**88**.**00** (250)	**75**.**60** (250)	44.80 (250)
HS	5.20 (250)	4.00 (250)	4.00 (250)	1.20 (250)	1.20 (250)	1.60 (250)
TS	0.00 (250)	0.00 (250)	0.00 (250)	0.00 (250)	0.00 (250)	0.00 (250)
TH	**62**.**40** (250)	**74**.**40** (250)	**79**.**20** (250)	**74**.**40** (250)	**67**.**60** (250)	38.80 (250)
TR	2.02 (248)	2.80 (250)	2.80 (250)	3.60 (250)	2.00 (250)	2.80 (250)
TSTR	0.00 (227)	0.00 (240)	0.00 (249)	0.00 (250)	0.00 (250)	0.00 (250)
THTR	35.94 (64)	40.78 (103)	34.18 (158)	35.52 (183)	24.12 (228)	25.51 (247)

### Carothers’ Test

The results of the Carothers test are presented in Table 7 and Web Table 6. In common with Leslie’s test, it is powerful at detecting heterogeneity in capture and also sensitive to trap-happiness and the combination of trap-happiness and transience. It is not sensitive to trap-shyness nor transience alone, or heterogeneity in survival. Unlike Leslie’s test, it retains a high power for a smaller sample size (see Web Table 6).

**Table 7:** Carothers’ test, $N=2000$ animals, percentage of significant results, high percentage of significant results in bold (> 50%).

Scenario	%
C1	5.20
C2	1.20
HC1	**100**.**00**
HC2	**100**.**00**
HC1t	**99**.**20**
HC2t	**100**.**00**
HCc1	**100**.**00**
HCc2	**87**.**60**
HCc3	**95**.**20**
HCc1F	**100**.**00**
HCc2F	**100**.**00**
HCc3F	**100**.**00**
HS	3.31 (242)
TS	0.00
TH	**100**.**00**
TR	1.20
TSTR	0.40
THTR	**99**.**20**

The number of applicable tests is 250 unless stated otherwise (indicated within brackets next to relevant number).

**Fig. 1:**
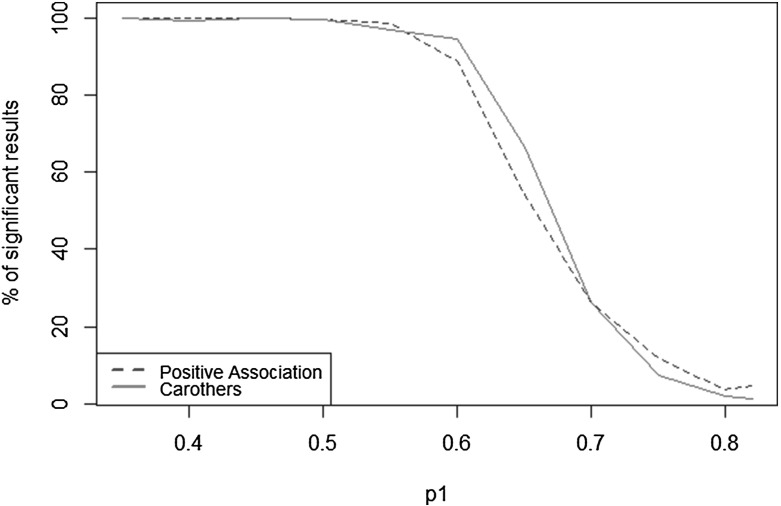
Power curves for the global test of positive association and Carothers’ test: percentage of significant results by capture probability in group 1 (the capture probability in group 2 is constant and set to 0.82) (Color figure online).

Both the Carothers test and the global test of positive association present similar characteristics and similar power to detect heterogeneity. We examine more closely the power of these two tests by using scenario HC1 and incrementally varying the capture probability of the low capture group by 0.05, so that the difference in capture probability between both groups decreases. The power curves obtained are presented in Fig. 1. Both tests display similar power: very high (close to 100 %) when the difference between the capture probabilities in each group is around 0.2 or more. The power of both tests decreases drastically for a smaller difference, with the Carothers test being slightly more powerful.

The outcome of the tests for each of the phenomena considered in this section is summarised in Table 8.

**Table 8:** Tests’ sensitivity to the phenomena examined, based on the simulation results for $N=2000$.

Test	Phenomena examined				
	Trap-happiness	Trap-shyness	Heterogeneity in capture	Heterogeneity in survival	Transience
Test of positive association (G)	✓	✗	✓ ✓	✗	✗
CJS diagnostic suite					
Test 3.SR	✗	✗	✓	✓	✓✓
Test 3.Sm	✗	✗	✓	✗	✗
Test 2.CT	✓✓	✓✓	✓	✗	✗
Test 2.CL	✗	✗	✓	✗	✗
Leslie test	✓✓	✗	✓✓	✗	✗
Carothers test	✓✓	✗	✓✓	✗	✗

A tick indicates that the test reacts to the phenomenon, and a double tick indicates that this phenomena was also the initial target of the test.

**Table 9:** Sandwich terns test results (NA for Leslie’s test if number of animals per group lower than 20, NA for positive association test if number of animals at given occasion lower than 30), d.o.f. denotes degrees of freedom, n denotes the number of animals used for the positive association test, significant results in bold.

Test		Sandwich terns results		
Positive association	Capture occasion	Test statistic	*n*	*p* value
	3	3.14	97	**0.001**
	4	3.55	115	<**0**.**001**
	5	3.27	121	**0.001**
	6	3.27	119	**0.001**
	7	1.18	89	0.12
Global positive association	–	5.03	182	**<0.001**

Diagnostic GOF	Component	Test statistic	d.o.f.	*p* value
	3.SR	127.27	8	**<0.001**
	3.Sm	38.13	17	**0.002**
	2.CT	140.02	7	**<0.001**
	2.CL	21.66	14	0.086
	Total	327.08	46	**<0.001**
	3.SR corrected	8.10	7	0.32
	2.CT corrected	8.58	6	0.20
	Total corrected	76.47	44	**0.002**

Leslie’s test	1st capture occasion	Test statistic	d.o.f.	*p* value
	1	200.92	96	**<0.001**
	2	NA	NA	NA
	3	NA	NA	NA
	4	NA	NA	NA
	5	NA	NA	NA
	6	66.71	35	**<0.001**

Carothers’ test		Test statistic	d.o.f.	*p* value
	–	569.08	304	**< 0.001**

## Sandwich Tern Application

The Sandwich tern dataset is formed from resightings of individuals ringed with small metal-rings engraved with unique numbers. Reading codes on this type of ring requires optimal conditions, i.e. proximity and good light. The ring-readings were not made inside the breeding colony of the Sandwich terns because the birds were nesting at a very high density inside a large colony of black-headed gulls *(Chroicocephalus ridibundus)*. Indeed, over the study period (2003–2012), 800–2400 pairs of Sandwich terns were breeding inside a black-headed gull colony holding 2500–6000 pairs. It was therefore impossible to access an observation hide inside or next to the Sandwich tern colony without causing extensive disturbance of a large number of gulls and terns. Instead the rings were read when the birds were roosting or preening in the immediate proximity of the colony. A number of large stones surrounded by shallow water constituted the preferred roosting and preening site for the terns (located approximately 300 m from the breeding colony). The major disadvantage of carrying out the resightings of the birds roosting on these stones was that not all of these individuals were actively engaged in a breeding attempt in the local colony. Thus some of the individuals were non-breeding birds that visited the colony, for example as prospectors, others were individuals that had stopped over before moving on to settle as breeders in another colony, and others were individuals that were visitors after having failed their breeding attempt in another colony. Due to the large array of possible behaviours of birds roosting on the stones, heterogeneity in capture was considered extremely likely. It was important to be able to detect its presence so as to use appropriate models to estimate survival and assess its temporal trends accurately. Several visits were made per year, but the dataset used for our analyses is annual in nature: it consists of all the individuals resighted at least once during the capture season. The dataset consisted of 1419 different individuals.

**Table 10:** Model fitting on the Sandwich terns dataset: time-dependent CJS with and without incorporating heterogeneity in capture (respectively, denoted by CJS and CJS (h)), maximum likelihood estimates and associated 95% confidence intervals.

Year	**CJS**		**CJS (h)**			
	$\phi _{t}$		$\phi _{t}$			
	MLE	95% CI	MLE	95% CI		
2003	0.78	(0.71, 0.84)	0.90	(0.70, 0.97)		
2004	0.77	(0.70, 0.83)	0.90	(0.76, 0.96)		
2005	0.84	(0.74, 0.90)	0.90	(0.77, 0.96)		
2006	0.81	(0.70, 0.89)	0.82	(0.72, 0.89)		
2007	0.66	(0.59, 0.73)	0.74	(0.65, 0.81)		
2008	0.89	(0.80, 0.94)	0.94	(0.84, 0.98)		
2009	0.72	(0.65, 0.77)	0.80	(0.73, 0.86)		
2010	0.80	(0.74, 0.85)	0.85	(0.78, 0.90)		

Year	$p_{t}$		$p_{t}$, group 1		$p_{t}$, group 2	
	MLE	95% CI	MLE	95% CI	MLE	95% CI
2004	0.66	(0.59, 0.73)	0.78	(0.69, 0.85)	0.28	(0.12, 0.52)
2005	0.74	(0.67, 0.80)	0.86	(0.78, 0.91)	0.17	(0.06, 0.39)
2006	0.53	(0.46, 0.60)	0.64	(0.56, 0.71)	0.06	(0.02, 0.18)
2007	0.53	(0.46, 0.60)	0.67	(0.58, 0.74)	0.05	(0.02, 0.13)
2008	0.58	(0.51, 0.65)	0.73	(0.65, 0.79)	0.01	(0.00, 0.10)
2009	0.61	(0.54, 0.67)	0.71	(0.64, 0.77)	0.12	(0.07, 0.20)
2010	0.62	(0.56, 0.68)	0.74	(0.67, 0.80)	0.04	(0.01, 0.12)
2011	0.76	(0.70, 0.82)	0.85	(0.79, 0.90)	0.15	(0.08, 0.26)

The results obtained from applying the tests to the Sandwich tern dataset, with 5% significance level, are presented in Table 9. For illustrative purposes, both the test per occasion and the global test were run for this dataset. The test of positive association yields a significant result at all occasions except for year 7, and the global positive association test also yields a significant result. This is indicative of trap-happiness or heterogeneity in capture. The diagnostic GOF tests indicate that the dataset presents transience and trap-happiness (see Web Table 7 for the directional components of Tests 2.CT and 3.SR). Also, Test 3.Sm yields a significant result whilst the Test 2.CL result is at the limit of significance. This suggests possible heterogeneity in capture. Leslie’s test result is NA in most cases due to sample size issues. Carothers’ test result is significant, suggesting trap-happiness or heterogeneity in capture.

Based on the ecological knowledge of the system and the obtained test results, we can conclude that the Sandwich tern dataset exhibits heterogeneity in capture. We fit a simple time-dependent CJS model as well as a model incorporating heterogeneity in capture using finite mixtures (Pledger et al. 2003), with two groups of animals. The models were fitted using program E-SURGE (Choquet et al. 2009b). Our aim was to assess whether survival estimates were affected when heterogeneity in capture was ignored. The parameter estimates obtained from fitting the two models are reported in Table 10. Note that due to the fact that *p* and $\phi$ are not estimable separately at the last occasion for the time-dependent model, we have not presented the estimates at the last occasion for either model. Pledger et al. (2010) cautiously recommended the use of the AIC for model comparison if there is no convergence problem and that there is no boundary estimate, which is the case here. The model including heterogeneity is found to be the best with $\mathrm{AIC}=5636.7$ (versus 5826.0 for the classic CJS time-dependent model). The results obtained reveals clearly contrasting capture probabilities: the average capture probability is 0.75 in one group and 0.11 in the other, with an estimated probability 0.59 of belonging to the group of highly capturable animals, with associated 95% CI (0.54, 0.65). The survival estimates displayed in Table 10 show that survival is underestimated when heterogeneity in capture is ignored. However, both models show an increased survival probability in year 2008; the year after gull culling was introduced.

The strong heterogeneity in this dataset led to questioning whether ring-reading should be continued for this study. Different selection criteria were applied to the data in order to focus on more homogeneous groups of birds, for instance by minimising the risk of including individuals that were not engaged in a breeding attempt in the study colony in the specific year of study. For example, one set of criteria included: resighted at least twice in the breeding season at an interval of at least 6 days with the first observation taking place in May. That reduced the dataset to 1483 observations of 756 individuals. However, applying the positive test per occasion still resulted in significant results at all occasions (except occasion 7); revealing that the reduced dataset still exhibited heterogeneity in capture. Note that the number of terns used for the test at occasion 3 was too low to use the normal approximation ($n=19$); hence, we used a nonparametric permutation test in order to derive an empirical p value.

## Discussion

We have proposed a test of positive association based on Goodman–Kruskal’s gamma to detect heterogeneity in capture. We chose the gamma measure since it is generally recommended for applications like ours, with a limited range in ranks and many ties (Siegel and Castellan 1988). Other measures of positive association using a correction for ties may also be used, such as Kendall’s Tau-b or Tau-c (Everitt 1992). Both these measures provided the same results as the test of positive association in terms of significant tests (results for some representative simulation scenarios are presented in Web Table 8); there was no difference between the results for Tau-b and those for Tau-c. Unlike other approaches to detect heterogeneity based on model comparison (Cubaynes et al. 2012), the test we propose does not need to specify anything about the other aspects of the model, nor a specific form of capture heterogeneity. This also means that the test gives no indication regarding the best way to model heterogeneity in the model fitting process. Our test of positive association is fairly easy to comprehend and compute unlike the alternative Carothers test; R code is provided as a supplementary file.

The global test of positive association is advantageous compared to the test per occasion since it provides a single result and is more powerful. However, it should be used only if little or no temporal variation is expected in capture probability. If this is not the case, and the sample size is sufficiently large, we recommend using the test per occasion. These recommendations are strengthened by additional simulations, run for an extreme case of time-dependence: there was no heterogeneity in capture but animals had capture probability $p=0.82$ over the first half of the experiment and $p=0.35$ over the second half. In this case, the test per occasion yields around 5% of significant results at each occasion whilst the global test yields 61.2% of significant results and hence demonstrates how heterogeneity in capture may be erroneously detected when there is none. Carothers’ test, on the other hand, is not affected by this (1.6% of significant results).

We have used simulation and an application to the Sandwich tern dataset in order to evaluate the test’s performance and have compared it to the existing diagnostic GOF tests as well as Leslie and Carothers’ tests for equal catchability. Our simulations have shown that none of the tests considered reacted to transience alone or heterogeneity in survival (apart from component 3.SR). The interpretation of the existing diagnostic GOF tests isn’t straightforward for detecting heterogeneity in capture. Indeed, when the components 2.CT, 2.CL, 3.SR and 3.Sm are all significant, this seems to indicate heterogeneity in capture. However, simulation has shown that there is relatively good power only when the sample size is very large and even in that case, there are situations where Test 2.CL lacks power. Therefore, if only some of the components are significant, the conclusion regarding heterogeneity in capture is not clear-cut. The Leslie and Carothers’ tests were both sensitive to violations of the assumption of equal recapture probability. Leslie’s test was shown to be impractical due to sample size issues whilst Carothers’ test was very powerful at detecting heterogeneity in capture, but also reacted strongly to trap-happiness.

The positive association test showed very high power at detecting heterogeneity in capture. Similarly to the Carothers test, it outperformed other approaches considered for detecting heterogeneity in capture. Whilst it reacted more strongly to heterogeneity in capture than to trap-happiness, it was also sensitive to this feature. Note that both these tests are conditional on survival, and their power to detect heterogeneity in capture will decrease with decreasing survival probability.

Trap-happiness is a complex phenomenon when considered relative to heterogeneity in capture. Indeed, trap-happiness increases the chances of concordant pairs whilst trap-shyness increases the chances of discordant pairs, especially for short sequences of previous and future encounters. Thus trap-happiness increases the chance of positive association, and trap-shyness diminishes it. As a result, trap-happiness may be confounded with heterogeneity whilst trap-shyness may mask it. The line between trap-happiness and heterogeneity in capture can be quite blurred: in real life, sampling may interact with behaviour in complex ways. For instance, when the cause of heterogeneous capture is the location, such as for the black-headed gulls (Prévot-Julliard et al. 1998), the birds may move between groups with low or high resighting propensity, and this will be statistically indistinguishable from trap-happiness.

When the test of positive association presented in this paper yields a significant result at any of the occasions, models accounting for heterogeneity in capture (and possibly trap-happiness, based on ecological expertise) should be considered at the model-building and model selection stage. Also, the causes of heterogeneity in capture should be investigated from a biological perspective. This may lead to the identification of individuals with different behavioural patterns or indicate whether an adjustment to sampling is necessary. For example, a high degree of heterogeneity in capture may indicate that a mixture of breeders and non-breeders is being sampled and in this case it might be advantageous to collect information on the breeding status of individuals. Even if imperfect, this information is very useful. If the group of interest is the breeders, the sampling process might be adjusted (e.g. sub-site or years selected to maximise the representation of breeders), or the data might be cleaned post hoc by applying strict criteria. Another possibility would be to create a smaller pilot study and adjust the sampling process accordingly on the final large study.

Due to the minimum number of capture occasions necessary to use our method, it has limitations for relatively short-lived organisms that are rarely observed more than three or four times in their life. In addition to this, the number of animals used for the test per occasion is relatively low compared to the original sample size; we therefore propose to derive an empirical p value from a nonparametric permutation test when the data are too sparse to use to normal approximation. Note also that by using the proportions of encounters, we have homogenised the information provided by all the animals; however, we lose the information on uncertainty provided by the denominator.

The test of positive association was explored for open populations in a CJS framework. But it could also be used in a context of population abundance estimation. Indeed the Jolly–Seber (JS) model, used to estimate abundance, assumes that unmarked and marked animals behave the same (McCrea and Morgan 2014, p.149). Applied to a JS context, if the test of positive association for marked animals yields a significant result, then the assumption of equal catchability is violated.

Further, the test of positive association and the Carothers test can both be used for closed populations. In this case, since the animals are known to be alive during the whole experiment, the whole encounter history becomes informative, including the information prior to the first capture occasion and after the last capture occasion. The test statistic proposed in this paper can then be straightforwardly adapted for this case.

## Supplementary Materials

Web tables and figures, referenced in Sect. 3, as well as the R code implementing the test of positive association for a capture history matrix, are available with this paper. The datasets are also available electronically with this paper.

## Electronic supplementary material

Below is the link to the electronic supplementary material.

Supplementary material 1 (pdf 138 KB)

Supplementary material 2 (R 19 KB)

Supplementary material 3 (csv 29 KB)

Supplementary material 4 (csv 15 KB)
