# Supplementary material for: A Test of Positive Association for Detecting Heterogeneity in Capture for Capture–Recapture Data
Source: J Agric Biol Environ Stat. 2017 Dec 11;23(1):1–19. doi: 10.1007/s13253-017-0315-4 (PMC6954010; doi:10.1007/s13253-017-0315-4)
Supplement: Supplementary file 1 — Supplementary material 1 (pdf 138 KB) [file 13253_2017_315_MOESM1_ESM.pdf]

Web-based Supplementary Materials for: A Test of Positive Association for Detecting Heterogeneity in Capture for Capture-Recapture Data

1 Web Appendix A: Simulation scenarios

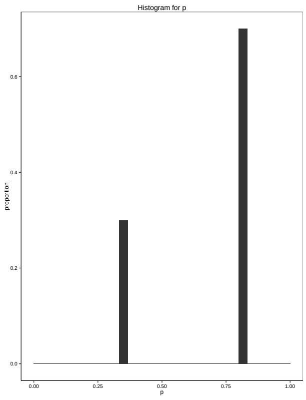

(a) HC1

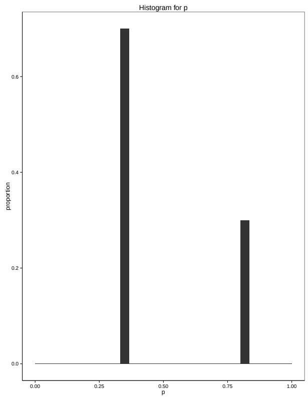

(b) HC2

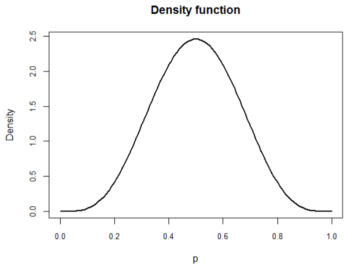

(c) HCc1

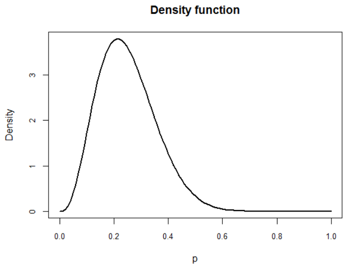

(d) HCc2

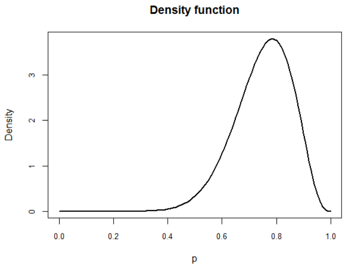

(e) HCc3

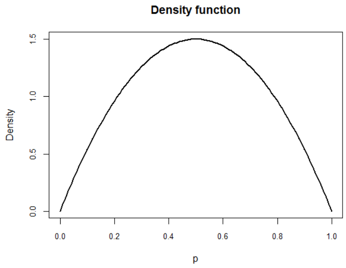

(f) HCc1F

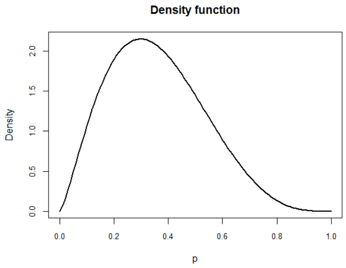

(g) HCc2F

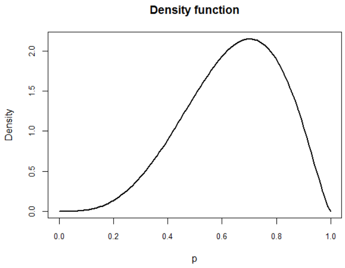

(h) HCc3F

Web Figure 1: Distribution of p for heterogeneity scenarios: histograms and density plots

Web Table 1: Table of percentiles for continuous heterogeneity in capture scenarios

Percentiles	10	20	30	40	50	60	70	80	90
HCc1	0.30	0.37	0.42	0.46	0.50	0.54	0.58	0.63	0.70
HCc2	0.12	0.16	0.19	0.21	0.24	0.27	0.30	0.34	0.39
HCc3	0.61	0.66	0.70	0.73	0.76	0.79	0.81	0.84	0.88
HCc1F	0.20	0.29	0.36	0.43	0.50	0.57	0.64	0.71	0.80
HCc2F	0.14	0.20	0.25	0.30	0.34	0.39	0.45	0.51	0.60
HCc3F	0.40	0.49	0.55	0.61	0.66	0.70	0.75	0.80	0.86

2 Web Appendix B: Simulation results for N=500 animals

Web Table 2: Percentage of significant results (number of applicable tests), test of positive association per occasion using Brown and Benedetti's asymptotic variance and 2 informative occasions, N=500, high percentage of significant results in bold (> 50%)

Scenario	3	4	5	6	7
C1	5.26 (57)	4.40 (250)	6.80 (250)	6.00 (250)	6.05 (248)
C2	6.59 (167)	6.80 (250)	5.60 (250)	6.40 (250)	7.60 (250)
HC1	**60.29** (136)	**84.00** (250)	**92.00** (250)	**90.00** (250)	**79.60** (250)
HC2	**81.40** (86)	**95.20** (250)	**98.00** (250)	**99.60** (250)	**97.60** (250)
HC1t	**84.62** (156)	**97.60** (250)	**92.80** (250)	**94.80** (250)	**90.40** (250)
HC2t	**84.62** (39)	**93.52** (247)	**99.20** (250)	**97.60** (250)	**89.92** (248)
HCc1	28.43 (102)	49.60 (250)	**54.40** (250)	**53.60** (250)	44.80 (250)
HCc2	0.00 (7)	22.58 (217)	24.90 (249)	20.40 (250)	17.98 (178)
HCc3	23.75 (160)	30.80 (250)	38.00 (250)	39.20 (250)	32.40 (250)
HCc1F	**75.00** (72)	**90.00** (250)	**93.20** (250)	**94.40** (250)	**88.80** (250)
HCc2F	**62.50** (24)	**54.44** (248)	**66.40** (250)	**66.40** (250)	**58.80** (250)
HCc3F	**50.39** (129)	**70.80** (250)	**79.60** (250)	**78.40** (250)	**70.80** (250)
HS	0.00 (25)	4.03 (248)	2.80 (250)	3.60 (250)	3.20 (250)
TS	2.48 (161)	0.80 (250)	3.20 (250)	0.80 (250)	0.80 (250)
TH	13.64 (66)	13.60 (250)	16.80 (250)	18.80 (250)	19.60 (250)
TR	NA (0)	9.86 (71)	5.51 (236)	6.02 (249)	4.00 (250)
TSTR	NA (0)	4.17 (72)	1.30 (231)	1.21 (248)	2.48 (242)
THTR	NA (0)	0.00 (15)	14.91 (114)	12.18 (156)	17.91 (67)

Web Table 3: Percentage of significant results (number of applicable tests), global test of positive association using Brown and Benedetti's asymptotic variance and 2 informative occasions, N=500, high percentage of significant results in bold (> 50%)

Scenario	% (N)
C1	6.40 (250)
C2	6.00 (250)
HC1	**98.00** (250)
HC2	**100.00** (250)
HC1t	**99.20** (250)
HC2t	**100.00** (250)
HCc1	**66.80** (250)
HCc2	29.60 (250)
HCc3	43.20 (250)
HCc1F	**98.80** (250)
HCc2F	**77.20** (250)
HCc3F	**91.20** (250)
HS	3.20 (250)
TS	1.60 (250)
TH	23.60 (250)
TR	6.40 (250)
TSTR	1.20 (250)
THTR	19.35 (248)

Web Table 4: Percentage of significant results (number of applicable tests), existing GOF components and corrected tests, N=500 animals, high percentage of significant results in bold (> 50%)

Scenario	3.SR	2.CT	2.CL	3.Sm	Total	3.SRC	2.CTC	TotalC
C1	4.00 (250)	5.60 (250)	0.00 (250)	5.20 (250)	4.00 (250)	5.20 (250)	6.00 (250)	3.60 (250)
C2	6.00 (250)	5.20 (250)	3.60 (250)	6.80 (250)	4.80 (250)	6.40 (250)	5.20 (250)	4.40 (250)
HC1	24.00 (250)	**90.00** (250)	29.60 (250)	16.40 (250)	**87.20** (250)	8.80 (250)	11.60 (250)	34.40 (250)
HC2	27.20 (250)	**82.80** (250)	8.40 (250)	21.60 (250)	**78.40** (250)	10.00 (250)	7.20 (250)	18.80 (250)
HS	**86.80** (250)	1.20 (250)	0.00 (225)	5.20 (250)	**51.20** (250)	10.80 (250)	0.40 (250)	2.40 (250)
TS	5.20 (250)	**100.00** (250)	0.40 (250)	6.80 (250)	**98.00** (250)	4.40 (250)	4.80 (250)	2.40 (250)
TH	4.00 (250)	**99.60** (250)	5.60 (250)	6.00 (250)	**93.60** (250)	3.60 (250)	10.00 (250)	8.00 (250)
TR	**100.00** (250)	0.80 (250)	0.82 (244)	4.40 (250)	**100.00** (250)	20.80 (250)	1.60 (250)	3.20 (250)
TSTR	**100.00** (250)	**87.20** (250)	0.40 (250)	6.80 (250)	**100.00** (250)	24.40 (250)	3.20 (250)	7.60 (250)
THTR	**100.00** (250)	**84.00** (250)	4.40 (250)	4.80 (250)	**100.00** (250)	3.60 (250)	4.80 (250)	4.40 (250)

Web Table 5: Percentage of significant results (number of applicable tests), modified Leslie's test, N=500 animals, high percentage of significant results in bold (> 50%)

1st release occasion	1	2	3	4	5	6
C1	0 (1)	NA (0)	NA (0)	0 (1)	0 (2)	0 (14)
C2	2.63 (76)	3.25 (123)	5.68 (176)	3.47 (202)	3.83 (235)	2.87 (244)
HC1	**82.14** (28)	**66.67** (42)	**67.62** (105)	**56.12** (139)	42.86 (189)	19.53 (215)
HC2	NA (0)	NA (0)	NA (0)	**100** (1)	**100** (1)	**100** (2)
HC1t	**92.22** (90)	**79.53** (127)	**81.77** (181)	**69.01** (213)	**57.85** (242)	35.12 (242)
HC2t	NA (0)	NA (0)	NA (0)	NA (0)	100 (3)	0 (2)
HCc1	NA (0)	NA (0)	100 (1)	NA (0)	0 (2)	0 (2)
HCc2	NA (0)	NA (0)	NA (0)	NA (0)	NA (0)	NA (0)
HCc3	28.57 (49)	17.33 (75)	15.89 (151)	18.67 (166)	11.26 (222)	6.96 (230)
HCc1F	**83.33** (6)	**100** (4)	**76.92** (13)	**63.64** (33)	49.02 (51)	24.05 (79)
HCc2F	NA (0)	NA (0)	0 (1)	0 (1)	40 (5)	8.33 (12)
HCc3F	**72.73** (11)	37.04 (27)	**53.85** (65)	32.76 (116)	23.64 (165)	14.29 (203)
HS	0 (7)	20 (15)	0 (22)	0 (46)	1.59 (63)	2.5 (80)
TS	0 (15)	0 (31)	0 (39)	0 (82)	0 (107)	0 (120)
TH	NA (0)	NA (0)	0 (4)	46.15 (13)	28.57 (28)	16.67 (48)
TR	NA (0)	NA (0)	0 (1)	0 (1)	0 (6)	12.5 (8)
TSTR	NA (0)	NA (0)	NA (0)	NA (0)	NA (0)	0 (5)
THTR	NA (0)	NA (0)	NA (0)	NA (0)	NA (0)	NA (0)

Web Table 6: Percentage of significant results (number of applicable tests), Carothers' test, N=500 animals, high percentage of significant results in bold (> 50%)

Scenario	% (N)
C1	10.40 (250)
C2	2.40 (250)
HC1	**95.20** (250)
HC2	**100.00** (250)
HC1t	**96.40** (250)
HC2t	**99.60** (250)
HCc1	**82.40** (250)
HCc2	**63.20** (250)
HCc3	46.80 (250)
HCc1F	**99.60** (250)
HCc2F	**94.40** (250)
HCc3F	**96.80** (250)
HS	4.00 (250)
TS	1.60 (250)
TH	**85.60** (250)
TR	2.80 (250)
TSTR	4.40 (250)
THTR	**58.40** (250)

3 Web Appendix C: Sandwich terns, results for directional components of Tests 2.CT and 3.SR

Web Table 7: Sandwich terns, existing GOF tests, directional components

**Test**	**test-statistic**	**p-value**
dir(3.SR)	10.92	<**0.001**
dir (2.CT)	-11.47	<**0.001**

4 Web Appendix D: Some results using Kendall's Tau-b and Tau-c

Web Table 8: Percentage of significant results (number of applicable tests), test of positive association based on Tau-b and Tau-c, N=2000

Scenario	Occasion	Tau-b	Tau-c
C1	3	4.00 (250)	4.00 (250)
	4	6.00 (250)	6.00 (250)
	5	2.80 (250)	2.80 (250)
	6	4.40 (250)	4.40 (250)
	7	3.20 (250)	3.20 (250)
C2	3	4.40 (250)	4.40 (250)
	4	3.60 (250)	3.60 (250)
	5	6.80 (250)	6.80 (250)
	6	6.40 (250)	6.40 (250)
	7	4.80 (250)	4.80 (250)
HC1	3	98.00 (250)	98.00 (250)
	4	100.00 (250)	100.00 (250)
	5	100.00 (250)	100.00 (250)
	6	100.00 (250)	100.00 (250)
	7	100.00 (250)	100.00 (250)
HC2	3	100.00 (250)	100.00 (250)
	4	100.00 (250)	100.00 (250)
	5	100.00 (250)	100.00 (250)
	6	100.00 (250)	100.00 (250)
	7	100.00 (250)	100.00 (250)
HCc2	3	37.20 (250)	37.20 (250)
	4	56.00 (250)	56.00 (250)
	5	59.60 (250)	59.60 (250)
	6	58.80 (250)	58.80 (250)
	7	44.80 (250)	44.80 (250)
HCc3	3	51.20 (250)	51.20 (250)
	4	76.40 (250)	76.40 (250)
	5	86.80 (250)	86.80 (250)
	6	89.20 (250)	89.20 (250)
	7	82.80 (250)	82.80 (250)
HCc1	3	76.40 (250)	76.40 (250)
	4	90.80 (250)	90.80 (250)
	5	98.40 (250)	98.40 (250)
	6	98.80 (250)	98.80 (250)
	7	94.40 (250)	94.40 (250)
TH	3	19.20 (250)	19.20 (250)
	4	30.40 (250)	30.40 (250)
	5	34.00 (250)	34.00 (250)
	6	45.20 (250)	45.20 (250)
	7	44.00 (250)	44.00 (250)
